# *De novo* small cell neuroendocrine carcinoma of the prostate with extremely elevated PSA and Gleason score 5 + 5: a case report

**DOI:** 10.3389/fonc.2025.1741030

**Published:** 2026-01-02

**Authors:** Yiwen Tang, Yuyang Cai, Xiaofeng Jiang, Ruoyu Qiao, Zhan Gao

**Affiliations:** 1 Department of Urology, Xiyuan Hospital, China Academy of Chinese Medical Sciences, Beijing, China; 2 Graduate School of Beijing University of Chinese Medicine, Beijing, China

**Keywords:** apalutamide, chemotherapy, Gleason grade, neuroendocrine prostate cancer, prostate-specific antigen, small cell carcinoma

## Abstract

**Objectives:**

The aims of this study were to report an exceptionally rare case of *de novo* small cell neuroendocrine carcinoma of the prostate (SCNEPC) presenting with unprecedented prostate-specific antigen (PSA) elevation and Gleason score 5 + 5, and to describe the remarkable treatment response achieved with multimodal therapy.

**Case:**

A 72-year-old man presented with PSA 982 ng/mL and extensive skeletal metastases. Prostate biopsy revealed mixed histology comprising small cell neuroendocrine carcinoma (Gleason 5 + 5 = 10) in seven cores and adenocarcinoma (Gleason 5 + 4 = 9) in three cores (T3bN1M1). The patient received six cycles of cisplatin–etoposide chemotherapy combined with goserelin and apalutamide. Post-treatment evaluation demonstrated profound biochemical response with PSA declining to 0.90 ng/mL (99.9% reduction), pro-gastrin-releasing peptide (ProGRP) decreasing to 75.7 pg/mL, and testosterone suppressed to castrate levels. Imaging confirmed substantial lesion regression with no new metastases. The patient experienced dramatic clinical improvement and remains alive with controlled disease under ongoing androgen deprivation therapy.

**Conclusion:**

This represents the first reported case of *de novo* SCNEPC with Gleason score 5 + 5, demonstrating that intensive multimodal therapy combining platinum-based chemotherapy with contemporary androgen receptor pathway inhibition can achieve profound and sustained responses in this aggressive variant typically associated with dismal outcomes. The substantial adenocarcinoma component may have contributed to the exceptional treatment response.

## Introduction

Neuroendocrine prostate cancer (NEPC) represents an uncommon but highly aggressive variant of prostate malignancy (1, 2). Treatment-induced NEPC (t-NEPC) emerges in approximately 20% of patients with metastatic castration-resistant prostate cancer following prolonged androgen deprivation therapy (1, 2). In contrast, *de novo* NEPC—present at initial diagnosis without prior hormonal manipulation—accounts for less than 2% of all prostate cancer cases (3).

Diagnosing *de novo* NEPC presents unique challenges. While patients with t-NEPC have documented disease progression following novel hormonal therapies, *de novo* cases lack this treatment history, potentially delaying recognition of neuroendocrine differentiation. Elevated neuroendocrine markers such as neuron-specific enolase (NSE) and pro-gastrin-releasing peptide (ProGRP) provide diagnostic clues (4). Both *de novo* and t-NEPC demonstrate significantly greater aggressiveness compared to conventional prostatic adenocarcinoma, with substantially poorer survival outcomes (5). Delayed or missed diagnosis profoundly impacts patient survival (6). Currently, no standardized therapeutic protocols exist for either variant.

Small cell carcinoma of the prostate, the most aggressive NEPC subtype, constitutes less than 1% of all prostate malignancies (7). These tumors typically present with advanced-stage disease characterized by visceral metastases and predominantly osteolytic bone lesions (7). The median overall survival is approximately 10 months, with a 5-year survival rate of only 12.6% (7). Cases presenting with exceptionally elevated prostate-specific antigen (PSA) levels combined with Gleason score 5 + 5 have not been previously reported.

Herein, we report an extraordinarily rare case of *de novo* metastatic mixed small cell neuroendocrine carcinoma of the prostate (SCNEPC) characterized by markedly elevated PSA (982 ng/mL), unprecedented Gleason score 5 + 5, and extensive bone metastases. The patient achieved remarkable clinical and biochemical responses following multimodal therapy combining cisplatin–etoposide chemotherapy with androgen deprivation and the novel androgen receptor inhibitor apalutamide.

## Case report

### Clinical presentation

A 72-year-old man presented to our institution in March 2025 following detection of elevated PSA during a routine community health screening. He had no significant past medical history or family history of malignancy. Although he denied lower urinary tract symptoms, the patient did report low back pain at initial presentation.

#### Initial laboratory findings

Total PSA: 982 ng/mL (reference: <4.0 ng/mL); free PSA: >50 ng/mL; free/total PSA ratio: 0.05; ProGRP: 148 pg/mL (reference: <63 pg/mL); NSE: within normal limits; carcinoembryonic antigen: within normal limits.

#### Physical examination

Digital rectal examination revealed Grade II prostatic enlargement with hard, nodular consistency, preserved borders, obliterated central sulcus, and no rectal bleeding.

### Imaging studies

#### Multiparametric prostate MRI

The prostate measured approximately 4.2 × 5.1 × 5.2 cm with imaging features highly suspicious for carcinoma. Left seminal vesicle invasion was evident, along with evidence of skeletal metastases.

#### Whole-body bone scintigraphy

Multiple areas of abnormally increased radiotracer uptake were identified throughout the axial and appendicular skeleton, including cervical, thoracic, lumbar, and sacral vertebrae with their posterior elements, bilateral ribs, sternum, pelvis, and left proximal femur. Several sites demonstrated features concerning for pathological fractures, particularly involving multiple ribs and the T8 vertebral body.

### Histopathological diagnosis

#### Specimen information

**Figure 1:**
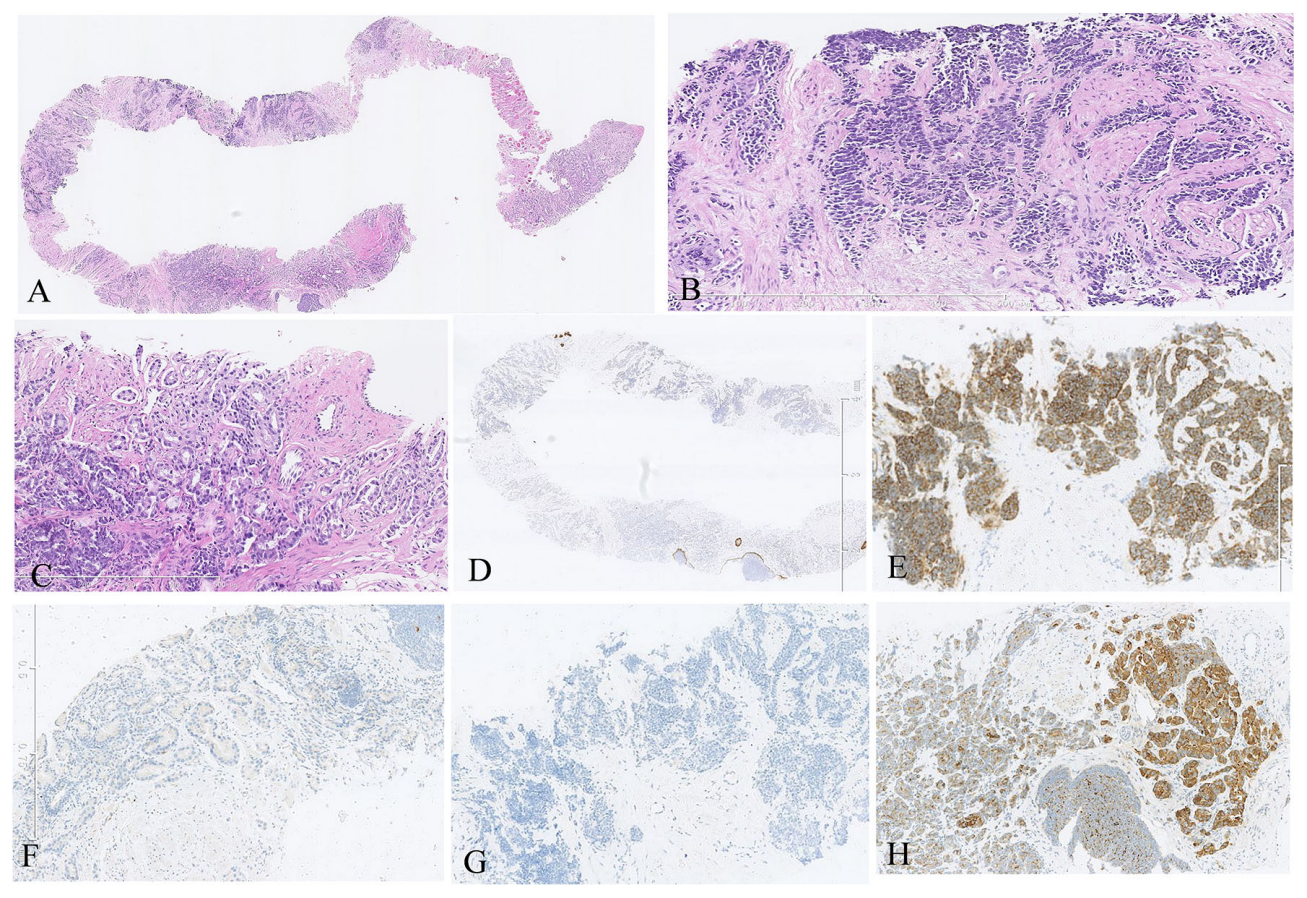
Histopathological and immunohistochemical examination of prostate biopsy tissue. **(A)** Hematoxylin and eosin (H&E) staining at 1.25× magnification showing the coexistence of prostatic adenocarcinoma and small cell neuroendocrine carcinoma within the same biopsy core. **(B)** H&E staining at 10× magnification demonstrating small cell neuroendocrine carcinoma with characteristic sheet-like arrangement, scant cytoplasm, finely granular chromatin, and high mitotic activity. **(C)** H&E staining at 10× magnification showing prostatic adenocarcinoma with small tubular and cribriform architecture, pale nuclei, and prominent nucleoli. **(D)** Immunohistochemical staining at 1.25× magnification showing negative 34βE12 expression in both prostatic adenocarcinoma and small cell neuroendocrine carcinoma. **(E)** Immunohistochemical staining at 5× magnification demonstrating strong positive synaptophysin expression in the small cell neuroendocrine carcinoma component. **(F)** Immunohistochemical staining at 5× magnification showing negative synaptophysin expression in the prostatic adenocarcinoma component. **(G)** Immunohistochemical staining at 5× magnification demonstrating negative PSA expression in the small cell neuroendocrine carcinoma component. **(H)** Immunohistochemical staining at 5× magnification showing moderate to strong positive PSA expression in the prostatic adenocarcinoma component.

Transperineal ultrasound-guided prostate biopsy obtained 10 cores. Histopathological examination identified the following (**Figure 1A**):

Seven cores (70%): Small cell neuroendocrine carcinoma (Gleason score 5 + 5 = 10)
Three cores (30%): Prostatic adenocarcinoma (Gleason score 5 + 4 = 9)

#### Microscopic morphology

The small cell component demonstrated classic morphology with sheets of small cells exhibiting scant cytoplasm, finely granular chromatin, and high mitotic activity (**Figure 1B**). The adenocarcinoma component showed cribriform architecture with prominent nucleoli (**Figure 1C**).

### Immunohistochemistry profile

Basal cell markers: 34βE12: negative (**Figure 1D**)
Proliferation marker: Ki-67: 70% positive
Prostatic markers: P504S: focally positive (approximately 10%)
Neuroendocrine markers: Synaptophysin: moderately positive in small cell component (**Figure 1E**), negative in adenocarcinoma (**Figure 1F**)
Chromogranin A: moderately positive; CD56: moderately positive
PSA: PSA staining: negative in small cell component (**Figure 1G**), positive in adenocarcinoma (**Figure 1H**)
**TNM staging:** T3bN1M1 (Stage IVB)

### Treatment protocol

Following multidisciplinary tumor board discussion, a combined treatment approach was implemented:

Chemotherapy (EP protocol):

Etoposide 130 mg intravenously (Day 1)
Cisplatin 60 mg intravenously (Days 1–2)
Cycle length: 21 days
Total cycles completed: 6 (April through July 2025)

Concurrent systemic therapy:

Androgen deprivation therapy: Goserelin 10.8 mg subcutaneously every 3 months (LHRH agonist)
Androgen receptor inhibitor: Apalutamide 240 mg orally daily
Bone-targeted therapy: Zoledronic acid 4 mg intravenously monthly

The selection of apalutamide was based on its favorable profile: minimal drug–drug interactions, no requirement for concurrent corticosteroids (unlike abiraterone), and lower incidence of fatigue compared to enzalutamide.

### Treatment response and follow-up

#### Clinical response

After the first chemotherapy cycle, the patient’s performance status improved substantially, with Eastern Cooperative Oncology Group (ECOG) score decreasing from 3 at baseline (attributed to pain from high tumor burden) to 2. Upon completion of all six cycles, the patient was pain-free with all analgesic medications discontinued. His urinary symptoms substantially improved, with International Prostate Symptom Score decreasing from 19 (severe) at presentation to 8 (mild) post-treatment.

#### Biochemical response (after six cycles)

Total PSA: 0.90 ng/mL (99.9% reduction from baseline)
Free PSA: 0.30 ng/mL
Free/total PSA ratio: 0.33
ProGRP: 75.7 pg/mL (48.7% reduction from baseline)
Testosterone: <0.09 nmol/L (castrate level achieved)
NSE and CEA: Remained within normal limits

#### Radiological response

**Figure 2:**
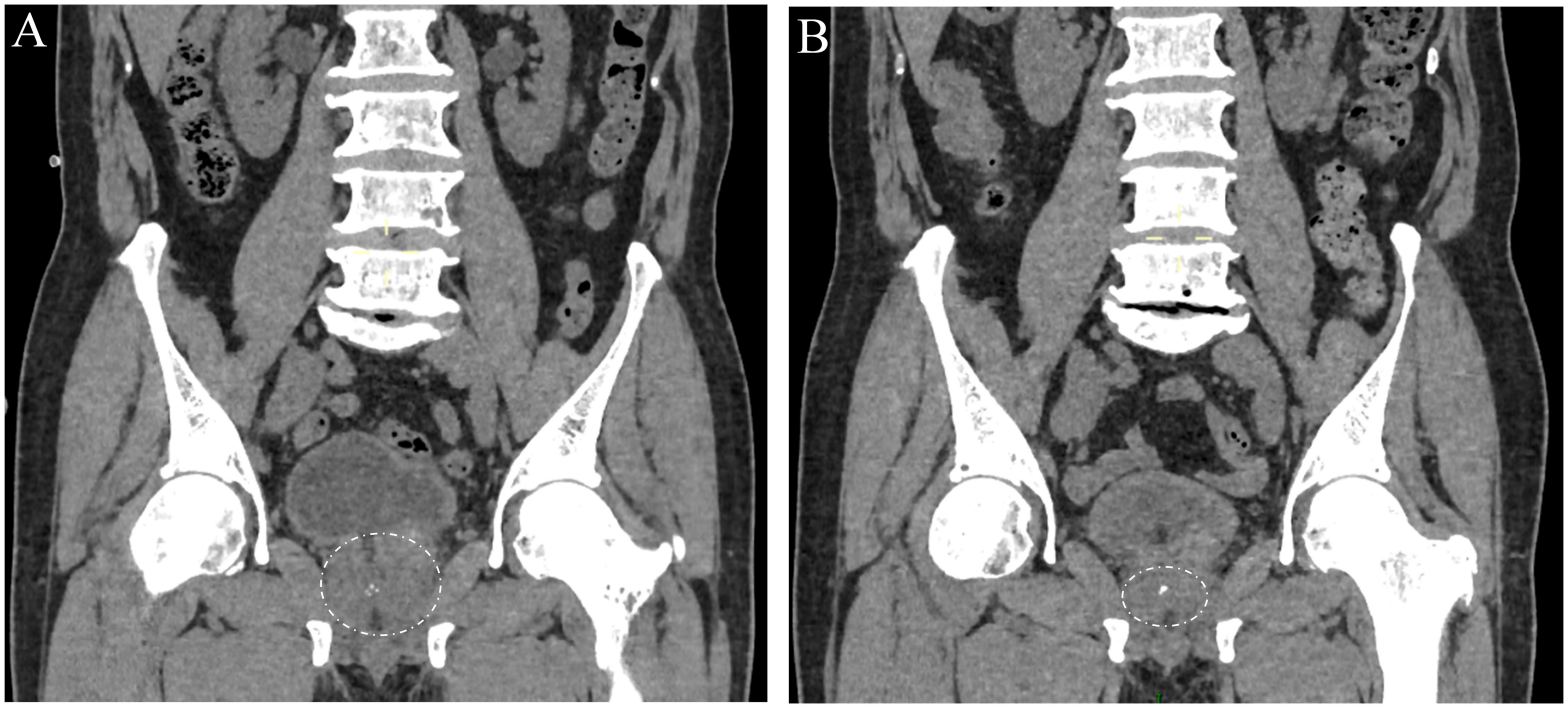
Computed tomography (CT) imaging of the pelvis without intravenous contrast at the level of the prostate during diagnosis and treatment stages. **(A)** Prior to treatment initiation. **(B)** Following completion of six cycles of chemotherapy combined with androgen receptor pathway inhibition. White circles indicate the boundary of the primary prostatic tumor, demonstrating substantial regression after therapy.

Urogenital CT imaging demonstrated substantial reduction in primary lesion dimensions (from 4.2 × 3.5 cm to significantly smaller, as indicated by the circle) with no new metastatic lesions identified (**Figure 2A**, **2B**). Follow-up assessment based on the patient’s clinical status (e.g., resolution of pain) and CT findings confirmed substantial regression of the primary lesion with no new metastatic sites, consistent with a positive therapeutic response.

#### Current status

At the time of manuscript preparation (approximately 8 months post-diagnosis), the patient continues to be under active surveillance with ongoing androgen deprivation therapy (goserelin plus apalutamide), demonstrating sustained disease control with excellent quality of life.

## Discussion

### Rarity and significance

This case represents the first documented report of *de novo* SCNEPC with Gleason score 5 + 5, the highest possible grade. SCNEPC is exceedingly rare, accounting for less than 1% of prostate malignancies (7). *De novo* presentation—without prior hormonal therapy—is even more uncommon, representing approximately 2% of NEPC cases (3, 7).

Our comprehensive literature review identified no prior cases with Gleason pattern 5 + 5 in the small cell component. This unprecedented finding underscores the exceptional biological aggressiveness of this tumor. The coexistence of high-grade adenocarcinoma (Gleason 5 + 4) in 30% of cores reflects the heterogeneity characteristic of aggressive prostate cancer.

### Extreme PSA elevation

The PSA level of 982 ng/mL represents one of the highest reported values in *de novo* SCNEPC. Pure small cell carcinomas typically demonstrate low or normal PSA due to loss of prostatic differentiation (4). The extreme PSA elevation in our case likely reflects the substantial admixed adenocarcinoma component, which retains PSA expression (confirmed by immunohistochemistry, **Figure 1H**).

PSA levels exceeding 100 ng/mL have become increasingly uncommon with widespread screening, occurring in only 7% of localized prostate cancer cases in contemporary series (8). PSA elevation to this degree portends the high likelihood of metastatic disease (9). The combination of extreme PSA (982 ng/mL), elevated ProGRP (148 pg/mL), and mixed histology guided our therapeutic approach toward primary systemic therapy rather than local treatment.

### Prognostic implications of Gleason grade group 5

The 2014 International Society of Urological Pathology (ISUP) classification stratified Gleason scores into five grade groups for improved prognostic discrimination (10). Patients within Grade Group 5 demonstrate markedly elevated risks of biochemical recurrence, distant metastasis, and cancer-specific mortality (11–13).

Contemporary evidence reveals prognostic heterogeneity within Grade Group 5. Surveillance, Epidemiology, and End Results (SEER) database analysis demonstrated that compared to Gleason 4 + 5, patterns 5 + 4 and 5 + 5 conferred 1.5-fold and 2.1-fold increased prostate cancer-specific mortality risk, respectively (14). This gradient emphasizes the extraordinary aggressiveness associated with primary Gleason pattern 5.

Despite this heightened risk, therapeutic approaches for Grade Group 5 generally parallel those for other high-risk categories. Meta-analysis data suggest that combining abiraterone acetate with androgen deprivation therapy and radiotherapy improves outcomes for node-positive or high-volume disease (15). However, data specific to Gleason 5 + 5 tumors remain limited, and applicability to neuroendocrine variants is unclear.

### Treatment rationale

Current management of SCNEPC extrapolates from small cell lung cancer (SCLC) treatment paradigms, given morphological and biological similarities. The 2022 National Comprehensive Cancer Network (NCCN) guidelines recommend platinum–etoposide combination as first-line therapy for limited-stage SCLC (16). For extensive-stage disease, the addition of PD-L1 inhibitors (atezolizumab or durvalumab) to platinum–etoposide is preferred (17).

However, outcomes for NEPC remain disappointing. A recent study of seven patients with NEPC treated with carboplatin–etoposide–atezolizumab reported a median progression-free survival of only 3.4 months and a median overall survival of 8.4 months (18). These dismal results underscore the profound therapeutic challenges.

Our approach incorporated several distinctive elements:

1. Chemotherapy backbone: Standard EP (etoposide–platinum) protocol aligned with SCLC guidelines and histological similarities between prostatic and pulmonary small cell carcinoma (19).
2. Novel hormonal therapy: Given the substantial adenocarcinoma component (30% of cores) and extreme PSA elevation suggesting retained androgen sensitivity, we added apalutamide to standard goserelin. Apalutamide offers several advantages: minimal drug–drug interactions, no requirement for concurrent corticosteroids (unlike abiraterone), lower pain incidence compared to enzalutamide, and proven efficacy in metastatic hormone-sensitive prostate cancer.
3. Bone-directed therapy: Given the extensive skeletal metastases with pathological fractures, zoledronic acid addressed skeletal-related events and may provide additional anti-tumor effects.

### Exceptional treatment response

The profound biochemical and clinical responses observed are unprecedented for this disease entity: PSA reduction: 99.9% decrease (from 982 to 0.9 ng/mL); clinical improvement: ECOG performance status improved from 3 to 0; symptom control: complete pain resolution, improved urinary function; radiological response: substantial lesion regression without new metastases; sustained remission: ongoing disease control at 8 months.

Literature review of reported *de novo* SCNEPC cases reveals median survival ranging from 20 days to 18 months, with no prior documentation of Gleason 5 + 5 disease (7). The exceptional response observed in this patient stands in stark contrast to the prognostic expectations typically associated with this disease entity and demonstrates the potential for achieving profound therapeutic benefit in this clinical context.

### Factors contributing to treatment success

Several factors may have contributed to the remarkable response:

1. Mixed histology: The substantial adenocarcinoma component (30% of cores) retained androgen sensitivity, explaining the dramatic PSA response to combined androgen deprivation and apalutamide. PSA-positive cells (**Figure 1H**) remained vulnerable to hormonal manipulation.
2. Chemosensitivity: *De novo* SCNEPC may demonstrate greater chemotherapy responsiveness compared to treatment-emergent disease, which typically develops through clonal selection and acquisition of resistance mechanisms under selective pressure of prolonged hormonal therapy.
3. Early intervention: Prompt initiation of multimodal therapy before extensive disease progression may have optimized treatment efficacy. Despite high tumor burden, the patient had not developed treatment-induced resistance mechanisms.
4. Synergistic mechanisms: Combined chemotherapy and hormonal manipulation likely provided complementary cytotoxic and cytostatic effects across heterogeneous tumor cell populations. Chemotherapy targeted rapidly dividing neuroendocrine cells, while hormonal therapy suppressed the adenocarcinoma component.

### Clinical implications

This case has several important clinical implications:

1. *De novo* SCNEPC can present with extreme PSA elevation when substantial adenocarcinoma components coexist. Extreme PSA elevation should not exclude consideration of neuroendocrine differentiation, particularly when disproportionate to clinical findings.
2. Mixed histology may predict differential treatment sensitivities. The presence of both small cell and adenocarcinoma components suggests potential benefit from combining chemotherapy targeting neuroendocrine cells with hormonal therapy targeting adenocarcinoma cells.
3. Novel androgen receptor pathway inhibitors warrant investigation in combination with chemotherapy for mixed histology NEPC. The synergistic effect observed with apalutamide suggests that routine incorporation of contemporary hormonal agents may improve outcomes beyond chemotherapy alone.
4. Aggressive upfront multimodal therapy can achieve remarkable responses even in highest-risk *de novo* NEPC. The profound and sustained response challenges nihilistic approaches to this disease entity.

### Limitations

The generalizability of our findings is limited by the nature of a single case report. Furthermore, cytokeratin 20 (CK20) staining was not performed; its inclusion in future diagnostic panels could help to further exclude rare metastatic mimics. Therefore, we plan to perform long-term follow-up of this patient to assess the durability of the treatment response and overall survival, which will provide further insights into the long-term management of this aggressive disease variant.

## Conclusion

We report an extraordinarily rare case of *de novo* mixed SCNEPC characterized by unprecedented Gleason score 5 + 5, extreme PSA elevation (982 ng/mL), and extensive metastases at presentation. This represents the first documented case of *de novo* SCNEPC with Gleason pattern 5 + 5.

Aggressive multimodal therapy combining cisplatin–etoposide chemotherapy with contemporary androgen receptor pathway inhibition (goserelin plus apalutamide) achieved profound and sustained biochemical and clinical responses. The 99.9% PSA reduction, substantial improvement in quality of life, and ongoing disease control at 8 months challenge the uniformly poor prognosis typically associated with this disease variant.

This case underscores the potential benefit of combining platinum-based chemotherapy with novel hormonal agents in *de novo* SCNECP with mixed histology. The substantial adenocarcinoma component may have contributed to the exceptional treatment response through retained androgen sensitivity. Further investigation is warranted to determine whether routine incorporation of androgen receptor pathway inhibitors with chemotherapy improves outcomes in this patient population.

## Data Availability

The raw data supporting the conclusions of this article will be made available by the authors, without undue reservation.
